# Effect of Graphene Oxide on the Acid Resistance of 3D-Printed Provisional Restorations Under Simulated Gastroesophageal Reflux Conditions

**DOI:** 10.3390/polym18070865

**Published:** 2026-04-01

**Authors:** Khanaphan Lebkrut, Atikom Surintanasarn, Tool Sriamporn, Awiruth Klaisiri, Taweesak Boonsod, Supachai Yanarueng, Kanoktip Boonkerd, Niyom Thamrongananskul

**Affiliations:** 1 Department of Prosthodontics, Faculty of Dentistry, Chulalongkorn University, Bangkok 10330, Thailand; 6571001932@student.chula.ac.th (K.L.); niyom.t@chula.ac.th (N.T.); 2 Division of Prosthodontics, College of Dental Medicine, Rangsit University, Pathumthani 12000, Thailand; 3 Division of Restorative and Esthetic Dentistry, Faculty of Dentistry, Thammasat University, Pathumthani 12120, Thailand; 4 Department of Chemistry, Faculty of Science, Kasetsart University, Bangkok 10900, Thailand; boonsod.sak@gmail.com; 5 Faculty of Public Health and Allied Health Sciences, Praboromarajchanok Institute, Nonthaburi 65000, Thailand; supachai.dds@gmail.com; 6 Department of Material Science, Faculty of Science, Chulalongkorn University, Bangkok 10330, Thailand; kanoktip.b@chula.ac.th; 7 Faculty of Dentistry, Burapha University, Chonburi 20131, Thailand

**Keywords:** 3D-printed provisional restorations, acid resistance, gastroesophageal reflux disease, graphene oxide, provisional dental materials, surface roughness, surface hardness

## Abstract

Recurrent acidic exposure in patients with gastroesophageal reflux disease (GERD) accelerates the degradation of provisional restorative materials, whereas approaches to enhance the acid resistance of 3D-printed restorations remain inadequately characterized. This study aimed to evaluate the effect of graphene oxide (GO) incorporation on the surface properties and acid resistance of 3D-printed provisional restorative materials under simulated gastroesophageal reflux conditions. GO was synthesized using the Hummers’ method and characterized by X-ray diffraction (XRD), Fourier transform infrared spectroscopy (FTIR), and Raman spectroscopy. XRD analysis demonstrated a pronounced shift in the characteristic peak (2θ) from 26° to 12°, consistent with an expansion of interlayer spacing after oxidation. FTIR confirmed the presence of oxygen-containing functional groups (hydroxyl, carbonyl, and epoxy), while Raman spectroscopy identified the characteristic D and G bands, confirming successful GO synthesis. Temporary Crown & Bridge resin (TC100) was modified with GO at six concentrations (0, 0.025, 0.05, 0.1, 0.5, and 1.0 wt %) using a planetary ball milling technique. Standardized 3D-printed specimens (n = 24 per group) were fabricated. Surface roughness and Vickers microhardness were measured before and after 45 h of immersion in simulated gastric acid (pH 2). Data were analyzed using one-way ANOVA and paired *t*-tests (α = 0.05). After acid exposure, the control group (0 wt % GO) exhibited significant surface deterioration, showing the highest surface roughness and a marked reduction in hardness (*p* < 0.05). Conversely, GO-modified groups demonstrated a concentration-dependent improvement in resistance to acid-induced degradation. The 0.5 wt % GO group showed the most favorable performance, maintaining both surface roughness and hardness with no significant difference from baseline values (*p* > 0.05). These findings indicate that GO incorporation enhances the surface integrity and acid resistance of 3D-printed provisional resins, with 0.5 wt % identified as the optimal concentration for minimizing acid-induced surface deterioration.

## 1. Introduction

Gastroesophageal reflux disease (GERD) is a chronic digestive disorder characterized by the backflow of stomach acid into the esophagus and sometimes into the oral cavity [1,2,3,4,5,6]. This condition affects a significant portion of the global population and can lead to various oral health complications, including dental erosion, mucosal irritation, and increased susceptibility to caries [7,8,9,10]. The presence of gastric acid in the mouth, particularly over extended periods, can compromise the integrity of both natural tooth structures and restorative dental materials [11,12,13]. In patients with GERD, the oral environment becomes more acidic than normal, posing a considerable challenge for the longevity and performance of dental restorations, especially provisional ones used during extended treatment phases.

Full mouth rehabilitation is a complex dental treatment approach aimed at restoring the function, esthetics, and health of a patient’s entire dentition [14]. Provisional restorative materials are indispensable in restorative dentistry that protect prepared tooth structures, maintain occlusal function, and support periodontal health. To perform these roles effectively, such materials must demonstrate comprehensive performance attributes, including superior mechanical properties [15,16,17,18,19], conformity with essential biological requirements, and sustained resistance to mechanical deterioration and chemical degradation. In this context, particular emphasis must be placed on the capacity of these materials to endure chemically aggressive oral environments, notably intrinsic acidic challenges associated with GERD, which represent a significant threat to material integrity, durability, and long-term clinical performance.

Provisional restorations used in full mouth rehabilitation can be fabricated using different materials and techniques, such as conventional bis-acrylic resins, polymethyl methacrylate (PMMA), and more recently, resin-based materials designed for digital workflows. Resin-based materials exhibit several advantageous properties, including high dimensional accuracy, superior surface resolution, fabrication efficiency, and compatibility with digital workflows [20]. With advancements in digital dentistry, 3D printing has become increasingly popular for fabricating provisional restorations due to its precision, efficiency, and ability to create complex geometries [20,21,22,23]. 3D-printed provisionals offer improved customization and faster production, making them highly suitable for full arch or full mouth cases. However, the resin materials currently used in 3D printing are still susceptible to degradation in low pH environments, which is a major concern in patients with GERD, whose oral cavity is routinely exposed to acidic conditions, and they are also associated with relatively high cost. Acidic exposure may compromise their mechanical integrity and clinical performance, highlighting the need for optimization; therefore, this study aims to improve their performance.

In response to these limitations, functional additives have been increasingly incorporated into dental polymer restorative materials to enhance their mechanical and physicochemical performance. Additives such as nanoparticles, fibers, and chemically active compounds have been reported to improve strength, wear resistance, and environmental stability, particularly under chemically challenging oral conditions [24]. Among these functional additives, GO, a derivative of graphene, is a carbon-based nanomaterial recognized for its distinctive physicochemical properties, including high mechanical strength, excellent thermal conductivity, antibacterial activity, and favorable biocompatibility [25,26,27]. In recent years, GO has attracted attention in the field of biomedical and dental materials due to its potential to reinforce polymers and improve their mechanical and functional performance [28]. Incorporating GO into dental materials has been shown to enhance flexural strength, hardness, and wear resistance, making it a promising additive in the development of next-generation restorative materials [27,29,30,31]. Additionally, graphene-based materials have demonstrated high chemical stability and resistance to degradation in acidic environments [32,33,34,35,36,37,38,39], making them particularly suitable for applications where exposure to low pH, such as in patients with GERD, is a concern [40,41,42]. Despite growing interest, its application in temporary or provisional restorations, especially those fabricated through 3D printing, remains underexplored.

While GO has been studied in permanent restorative materials such as composites, adhesives, and cements, there is a significant lack of data regarding its use in provisional materials, particularly in the context of 3D-printed restorations. No current literature thoroughly evaluates how GO incorporation affects the surface properties and acid resistance of 3D-printed provisional restorations subjected to acidic environments like those present in GERD. This knowledge gap limits the advancement of material development for patients requiring long-term temporization under high-risk oral conditions, such as those with GERD undergoing full mouth rehabilitation. Dental resin materials are susceptible to degradation under acidic conditions, resulting in increased surface roughness and reduced hardness [43,44]. Surface roughness and Vickers microhardness measurements are commonly used to assess changes in their surface characteristics following acidic exposure.

This study aims to evaluate the effect of GO incorporation on the surface properties and acid resistance of 3D-printed provisional restorations under simulated gastroesophageal reflux conditions, and to compare the surface alterations between immersed specimens and their respective non-immersed baseline controls within each concentration group. By simulating an acidic environment representative of the oral conditions in patients with GERD, this study evaluates whether GO-enhanced materials exhibit improved performance and durability while utilizing a more cost-effective resin system. The incorporation of GO enables performance comparable to higher-cost commercial resin materials, underscoring its potential as an economical alternative for dental applications. These findings could contribute to the development of more durable provisional materials for high-acidic oral environments and establish a foundation for further investigation into the broader application of nanomaterials in both temporary and permanent dental restorations. The first null hypothesis of this study was that varying the concentration of GO incorporated into 3D-printed provisional restorative materials would not result in statistically significant differences in surface roughness and surface hardness after immersion in a simulated gastroesophageal reflux environment. The second null hypothesis was that exposure to the simulated gastroesophageal reflux environment would not result in statistically significant alterations in the surface properties of the materials within each GO concentration group when compared to their respective non-immersed baselines.

## 2. Materials and Methods

The methodology of this study was systematically divided into three distinct phases to investigate the influence of GO incorporation on the surface properties of 3D-printed provisional restorative materials under simulated acidic conditions. The first phase involved the synthesis of GO using the Hummers’ method [45], aimed at producing high-purity GO suitable for integration into dental resin matrices. In the second phase, the synthesized GO was incorporated into the 3D-printed provisional restorative materials, followed by the fabrication of standardized specimens utilizing additive manufacturing technology. The third phase focused on the evaluation of key material properties, including surface roughness and surface hardness under both neutral and simulated acidic conditions. This comprehensive approach enabled the assessment of the surface characterization and acid resistance of GO-modified 3D-printed provisional materials. All experimental procedures were performed by a single trained and calibrated operator to minimize operator-related variability.

### 2.1. Materials Preparation

The materials utilized in this study, along with their respective compositions and manufacturers, including those employed for GO synthesis, gastric acid preparation, and specimen preparation, are presented in Table 1.

### 2.2. Synthesis of GO

**Figure 1:**
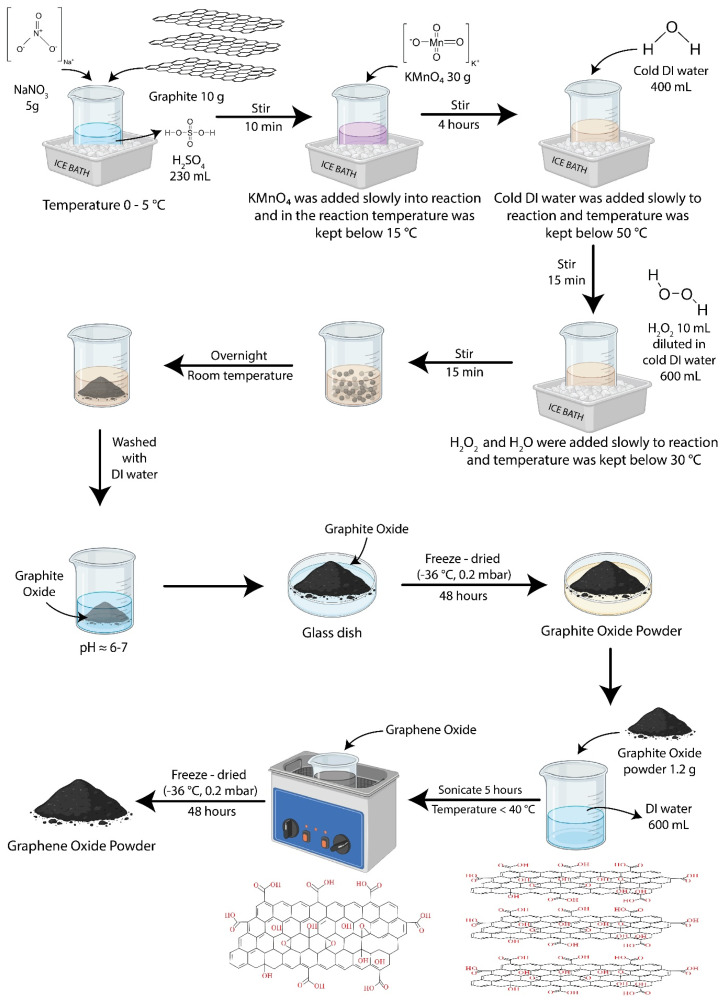
Process flow diagram illustrating the sequential steps involved in the synthesis of GO.

GO was synthesized from natural graphite powder based on the Hummers’ method [45]. Sulfuric acid (H_2_SO_4_, 230 mL) was precooled to 0–5 °C under continuous magnetic stirring. Natural graphite powder (10 g) and sodium nitrate (NaNO_3_, 5 g) were then gradually added to the cooled acid with constant agitation, ensuring homogeneous dispersion and preventing localized overheating, and the mixture was maintained under magnetic stirring for 10 min. Subsequently, 30 g of potassium permanganate (KMnO_4_) was slowly added while maintaining the temperature below 15 °C under constant stirring at 250–300 rpm to control the exothermic reaction. After stirring for 4 h, cold deionized water (400 mL) was added to the mixture while keeping the temperature below 50 °C. Next, 30% hydrogen peroxide (H_2_O_2_, 10 mL) and cold deionized water (600 mL) were carefully added until a yellow-brown coloration was observed; this observation suggested successful oxidation and exfoliation of the graphite flakes. The resulting mixture was stirred continuously for 15 min and subsequently allowed to stand at ambient temperature overnight to ensure complete oxidation and facilitate the full precipitation and stabilization of graphite oxide. The calculated pH value of the solution was determined to be 0. Subsequently, the graphite oxide powder was thoroughly dispersed in deionized water, and the pH of the suspension was carefully adjusted to 6–7. The graphite oxide precipitate obtained from this procedure was subsequently freeze-dried at –36 °C and 0.2 mbar for 48 h, facilitating the removal of residual moisture while preserving the intrinsic layered structure of the material, and ultimately yielding a dry and stable graphite oxide powder. Subsequently, the graphite oxide powder (1.2 g) was thoroughly dispersed in deionized water (600 mL) and subjected to sonication for 5 h under temperature-controlled conditions using an ice bath. This procedure ensured uniform exfoliation of the graphite oxide layers while preventing thermal degradation or aggregation, ultimately yielding a stable, fully single-layered GO suspension. Finally, the single-layered GO suspension was subjected to vacuum-assisted freeze-drying at –36 °C and 0.2 mbar for 48 h, facilitating complete removal of residual moisture and yielding a dry, stable GO powder suitable for subsequent applications (Figure 1). The synthesized GO was stored in a desiccated environment using silica gel to minimize moisture exposure.

### 2.3. Characterization of GO

X-ray diffraction (XRD) analysis was performed using a Bruker D8 Discover diffractometer (Bruker AXS GmbH, Karlsruhe, Germany) with Cu Kα radiation (λ = 1.5406 Å, 40 kV, 40 mA) over a 2θ range of 5–60° at a scanning rate of 2°/min to evaluate the crystalline structure and interlayer spacing of GO. The average number of graphene layers was estimated using the Debye–Scherrer equation to assess the degree of exfoliation.

$$t=\frac{0.89\lambda }{\beta \;Cos\theta }\;and\;n=\frac{t}{d}$$

where: *t* is the crystallite size; *λ* is the X-ray wavelength; *β* is the full width at half maxima (FWHM) in radians; *θ* is the Bragg diffraction angle; *n* is the number of layers, *d* is the interlayer spacing.

Fourier transform infrared (FTIR) spectroscopy Nicolet™ iS50 FTIR spectrometer (Thermo Fisher Scientific, Madison, WI, USA) was performed over 4000–400 cm^−1^ at a resolution of 4 cm^−1^ with 16 scans to investigate the chemical structure and identify the functional groups present in the synthesized materials. Characteristic O–H, C=O, C–OH, and C–O absorption bands confirmed the presence of oxygenated functionalities and the successful oxidation of graphite to GO [46].

Raman spectroscopy analysis was conducted using a Confocal Raman Microscope (XploRA™ PLUS, HORIBA France SAS, Loos, France) with a 532 nm excitation laser to characterize the structure of GO. The G band corresponds to graphitic carbon structures, whereas the D band is attributed to structural defects and partially disordered graphitic domains [47]. The characteristic D (~1350 cm^−1^) and G (~1580 cm^−1^) bands were identified, and the I_D_/I_G_ ratio was used to assess structural disorder and oxidation, with higher ratios indicating increased defects and successful oxidation of graphite to GO [46].

### 2.4. Sample Size Calculations

**Figure 2:**
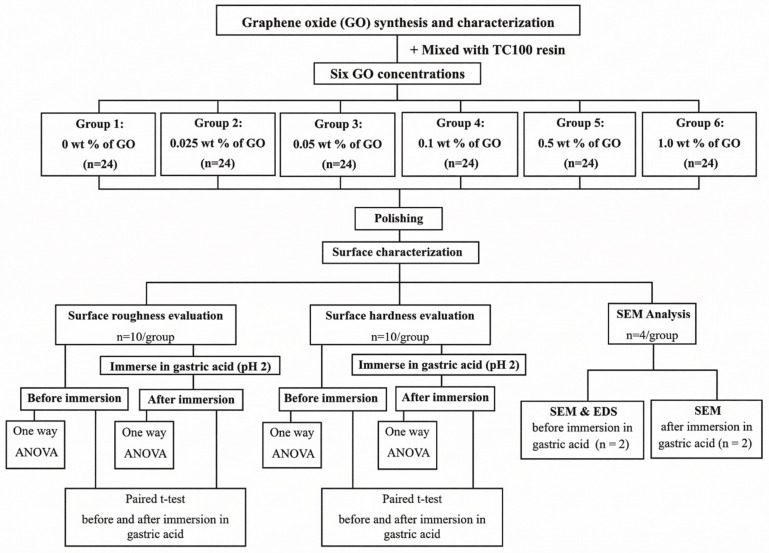
A flowchart of the experimental procedure for evaluating surface properties and acid degradation of GO-reinforced resin.

G*Power software (Version 3.1.9.7, Heinrich Heine University Düsseldorf, Düsseldorf, Germany) was used to determine the required sample size for this study. The parameters used in the analysis included an effect size of f = 0.43 (derived from an in vitro pilot study), a significance level of α = 0.05, and power (1 − β err prob) = 0.95. Based on these inputs and 6 experimental groups, the calculated total sample size required was 114 specimens. Consequently, the sample size was rounded up to 24 specimens per group to ensure a balanced design, resulting in a total of 144 specimens for the investigation. Within each group, 10 specimens were designated for surface hardness testing and the remaining 10 for surface roughness measurement. Additionally, four specimens per group were allocated for SEM analysis (Figure 2).

### 2.5. Preparation of GO-Modified Resin Specimens

Specimens (10 mm diameter, 2.0 ± 0.2 mm height) were designed using Chitubox^®^ Basic V2 software (CBD-Tech, Shenzhen, China) and fabricated with a Phrozen Sonic Mini 8K LCD 3D Printer (Phrozen Tech Co., Ltd., Hsinchu City, Taiwan) [48], operating at a wavelength of 405 nm to cure each printed layer, ensuring standardized and reproducible fabrication. The printing parameters were set to an exposure time of 3 s, bottom exposure time of 30 s, lifting distances of 6 mm, lift speeds of 80 and 60 mm/min for normal and bottom layers, respectively, and a retract speed of 150 mm/min.

In this experimental study, TC 100 Temporary Crown and Bridge Resin (Shenzhen Esun Industrial Company, Shenzhen, China) served as the polymer matrix. GO was incorporated at six different concentrations (0, 0.025, 0.05, 0.1, 0.5 and 1.0 wt %) into the resin and uniformly dispersed using a planetary ball milling process [49]. To ensure homogeneous dispersion of the nanofiller, the mixture was processed using a planetary ball milling technique at a rotational speed of 500 rpm for a total duration of 15 min under ambient temperature. To minimize heat buildup and prevent premature polymerization, an intermittent milling protocol was employed, consisting of 5 min milling cycles followed by 2 min cooling intervals. The temperature of the milling chamber was strictly monitored and maintained below 30 °C to preserve the physicochemical stability of the resin system [50]. Upon completion of the 3D printing process, the specimens underwent post-processing, which involved immersion in 99% isopropyl alcohol (IPA) for 2 min in an ultrasonic cleaning system to effectively eliminate any residual uncured resin. Following cleaning, the specimens were air-dried for 10–15 min to remove residual solvent and then post-cured under 405 nm light irradiation for 10 min at 20–25 °C to achieve complete polymerization and material stabilization.

The surfaces of all specimens were standardized through a sequential polishing protocol using an automated polishing system (EcoMet30, Buehler, Lake Bluff, IL, USA) under continuous water-cooling. Specimens were initially polished using silicon carbide abrasive papers of progressively increasing grit sizes (120, 240, 320, 600, 800, and 1000-grit) (3M Wetordry Abrasive Sheet, 3M, Maplewood, MN, USA) at a rotational speed of 300 rpm for 300 s per grit under a constant load of 10 N. Subsequent surface refinement was achieved using a diamond cutting wheel, followed by sequential polishing with MetaDi^®^ II Diamond Paste (Buehler, Lake Bluff, IL, USA) and Aka-Paste Mono 1 µm monocrystalline diamond paste (Akasel, Roskilde, Denmark). This multi-step finishing protocol yielded a highly smooth and lustrous surface, closely resembling the polished surface characteristics of provisional crown restorations employed in clinical practice. The specimens were standardized to a final dimension of 10 mm in diameter and 2 mm in thickness, with dimensional accuracy verified using a precision digital caliper (Dentaguage 1; Erskine Dental, Erskine, UK). Following polishing, specimens underwent ultrasonic cleaning (Ultrasonic Cleaner VI; Yoshida Dental Trade Distribution Co., Tokyo, Japan) in distilled water for 10 min to eliminate any surface debris, were air-dried, and subsequently stored at room temperature under dry conditions until further experimental analysis.

### 2.6. Acidic pH Immersion Protocol

A simulated gastric acid solution was prepared in accordance with the method proposed by Hunt and McIntyre [51] to mimic the clinical conditions of gastric fluid. The solution was prepared by dissolving 2 g of NaCl and 3.2 g of pepsin in 1000 mL of distilled water, followed by the addition of 7 mL of HCL. The solution was thoroughly mixed until fully homogenized, resulting in a final pH of approximately 2, thereby simulating the gastric environment, with pH measurements conducted every 24 h to maintain experimental consistency. For each temporary material, the 144 specimens were randomly divided into six groups (n = 24 per group) in different concentration GO (0, 0.025, 0.05, 0.1, 0.5 and 1.0 wt %). To simulate the effects of prolonged acidic exposure, all specimens from each material group were fully immersed in individual Petri dishes containing the prepared gastric acid solution. The samples were subsequently incubated in a controlled environment at 37 °C using an incubator (Thermo Fisher Scientific, Waltham, MA, USA) for a predetermined period of 45 h [52]. This exposure duration corresponds to approximately 6 months of clinical exposure to gastric acid in patients with conditions such as bulimia [52]. After each exposure period, specimens were carefully rinsed with deionized water to remove residual acid, air-dried at room temperature, and stored under dry conditions until further testing. Surface characterization was conducted at baseline (prior to immersion) and following 45 h of exposure to the acidic medium across all GO concentrations and the control group.

### 2.7. Surface Characterization

The specimens were randomly allocated for the assessment of surface roughness and surface hardness.

#### 2.7.1. Surface Roughness

Surface roughness was evaluated using a contact stylus profilometer (Talyscan 150, Taylor Hobson, Leicester, UK). The measurements were conducted with a stylus tip of 2 µm radius, applying a contact force of 0.7 mN. The probe traversed a 5 mm path along the center of each specimen at a scanning speed of 0.5 mm/s, with a cut-off value set at 0.8 mm to determine the arithmetic mean roughness (Ra). For each specimen, the Ra values were obtained from ten parallel scans and averaged to ensure accuracy. Each specimen served as its own control, with evaluations conducted before and after immersion to allow for paired comparisons.

#### 2.7.2. Surface Hardness

Surface microhardness was evaluated using a Vickers microhardness tester (FM-810, Future-Tech Corp., Kanagawa, Japan). In accordance with the ISO 6507-1:2023 standard [53], three indentations were made on each specimen surface, equally distributed along a circular path, ensuring a minimum distance of 1 mm between adjacent indentations. A test load of 50 gf (HV 0.05) was applied with a dwell time of 15 s. The diagonal lengths of each indentation were measured using an optical microscope at 20× magnification, and the Vickers hardness number (VHN) was calculated using the formula: HV = 1.854 P/d^2^, where HV is the hardness value in kg/mm^2^, P is the applied load in kgf, and d is the mean diagonal length in mm. The average of the ten measurements was reported as the final hardness value for each group. Evaluations were conducted at baseline (before immersion) and after the 45 h acid exposure to allow for paired statistical comparisons.

#### 2.7.3. Scanning Electron Microscopy (SEM) and Energy-Dispersive X-Ray Spectroscopy (EDS) Analysis

For SEM-EDS analysis, two specimens were randomly chosen from each group prior to immersion in gastric acid. For SEM analysis, two specimens were randomly selected from each group following immersion in gastric acid. The specimens were air-dried at room temperature for 24 h prior to examination. Subsequently, they were sputter-coated with a thin layer of gold using a sputter coater (JFC-1200, JEOL, Tokyo, Japan) to enhance electrical conductivity and minimize surface charging artifacts. Surface morphology and elemental composition were evaluated using a scanning electron microscope equipped with EDS (Quanta 250, FEI, Hillsboro, OR, USA). The SEM was operated in secondary electron (SE) mode at an accelerating voltage of 10 kV and a working distance of 10 mm. Micrographs were captured at a magnification of 10,000× to examine the detailed surface topography of the specimens.

### 2.8. Statistical Analysis

Statistical analyses were performed using IBM SPSS Statistics for Windows, Version 29.0 (IBM Corp., Armonk, NY, USA). All quantitative data are expressed as mean ± standard deviation (SD). The normality of data distribution and homogeneity of variances were verified using the Shapiro–Wilk test and Levene’s test, respectively. One-way ANOVA was conducted to evaluate the differences in surface roughness and surface hardness among the different GO concentrations after acid immersion. When significant differences were detected, Tukey’s Honestly Significant Difference (HSD) test was applied for post hoc multiple comparisons.

To compare surface properties between baseline (non-immersed) and post-immersion measurements within each GO concentration group, paired *t*-tests were employed for normally distributed data. All statistical analyses were performed at a 95% confidence level, and statistical significance was established at *p* < 0.05.

## 3. Results

### 3.1. Characterization of GO

Oxidation of graphite introduced oxygen-containing functional groups, including hydroxyl, carboxyl, and epoxy groups, onto its surface, thereby weakening interlayer interactions and facilitating exfoliation of graphite into individual GO sheets. In addition, the presence of polar oxygenated groups increased the hydrophilicity of GO, which promoted its dispersion stability in aqueous solution. To ensure the suitability of the synthesized GO for subsequent experimental applications, comprehensive characterization was performed in advance. Structural and chemical characteristics were systematically investigated using XRD, FTIR, and Raman spectroscopy.

**Figure 3:**
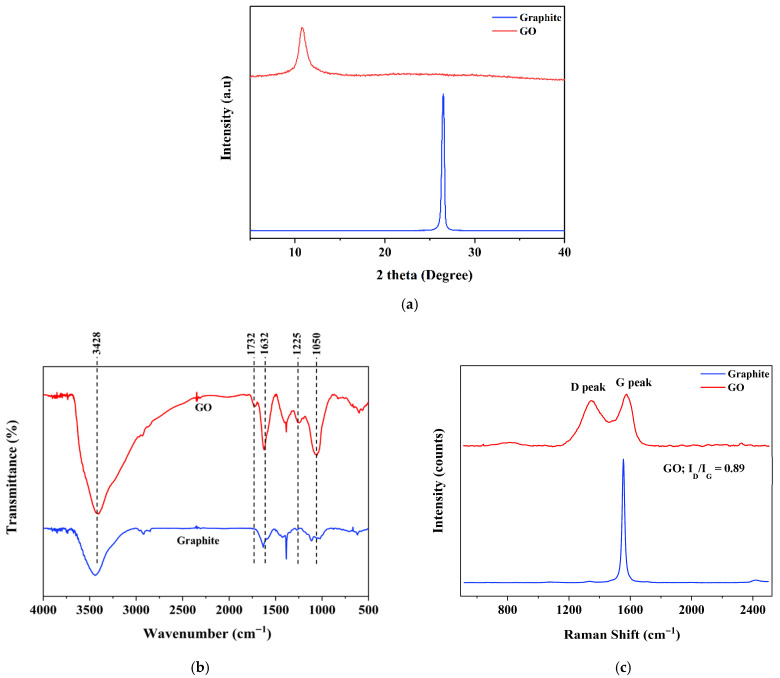
Characterization of GO: (**a**) X-ray diffraction (XRD) pattern of GO; (**b**) Fourier transform infrared (FTIR) spectrum of GO; (**c**) Raman spectrum of GO.

The XRD pattern of the GO (Figure 3a) exhibits a dominant diffraction peak at 2θ ≈ 12°, corresponding to an interlayer spacing (d-spacing) of approximately 0.77 nm, as calculated using Bragg’s law with Cu Kα radiation (λ = 1.5406 Å). In contrast, graphite shows a characteristic diffraction peak at 2θ ≈ 26°, corresponding to a d-spacing of approximately 0.37 nm. The shift in the diffraction peak from 26° to 12° clearly indicates a substantial expansion of the interlayer distance after oxidation. This structural transformation is attributed to the successful insertion of oxygenated functional groups, such as hydroxyl, epoxy, and carboxyl groups, into the graphitic lattice. The increase in interlayer spacing is a well-recognized characteristic of GO and strongly supports the effective oxidation of graphite.

The FTIR spectra of graphite and GO are presented in Figure 3b. Both spectra exhibited absorption bands at approximately 1632 cm^−1^ and 3428 cm^−1^, assigned to C=C and O–H stretching vibrations, respectively. However, the O–H band observed in GO was more than that of graphite, indicating the substantial enrichment of hydroxyl-containing species after oxidation. This enhancement is attributed to the introduction of oxygenated functionalities during oxidation. In addition, the FTIR spectrum of GO displayed new characteristic absorption peaks at 1732 cm^−1^, 1225 cm^−1^, and 1050 cm^−1^. These bands were assigned to C=O stretching vibrations of carbonyl groups, C–O stretching associated with hydroxyl-containing carboxylic functionalities, and alkoxy C–O stretching vibrations, respectively [54,55,56,57]. The appearance of these oxygen-related bands, absent in graphite, provides strong evidence of successful graphite oxidation and the formation of GO. Collectively, these spectral changes confirm the incorporation of hydroxyl, epoxy, carboxyl, and carbonyl functional groups into the graphite structure, which is consistent with the characteristic chemical features of oxidized graphene-based materials.

The Raman spectrum of graphite exhibited a prominent G band at approximately 1590 cm^−1^, which is attributed to the in-plane vibration of sp^2^-hybridized carbon atoms within the graphitic structure. In contrast, the Raman spectrum of GO displayed both the G band at approximately 1590 cm^−1^ and a distinct D band at approximately 1350 cm^−1^, with an I_D_/I_G_ ratio of 0.89 (Figure 3c). The appearance of the D band and the increase in the I_D_/I_G_ ratio indicate the generation of structural defects and a reduction in the structural ordering of the carbon framework after oxidation [54,58]. Chemical oxidation disrupts the extended conjugated sp^2^ carbon network of graphite, leading to partial destruction of the regular graphitic structure and increased disorder. Therefore, the Raman results provide strong evidence for the successful oxidation and structural modification of graphite into GO. These findings are also consistent with the XRD and FTIR results, which collectively confirm the formation of the GO structure. More importantly, the enlarged gallery spacing weakens interlayer van der Waals interactions, thereby reducing layer restacking and facilitating the exfoliation of GO into thinner sheets. Such structural evolution is crucial, as it directly influences the dispersibility, surface activity, and subsequent interfacial interactions of GO in composite or resin-based systems.

### 3.2. Surface Characterization

#### 3.2.1. Surface Roughness

Table 2 summarizes the surface roughness (Ra, µm) values of TC100 incorporated with different concentrations of GO before and after immersion in a simulated gastroesophageal reflux environment. Prior to acid immersion, the surface roughness values across all experimental groups ranged from 0.0226 to 0.0294 µm. Statistical analysis indicated no significant differences among the control group and GO-modified groups at any concentration (*p* > 0.05), suggesting that GO incorporation did not significantly influence the initial surface characteristics of the material.

Following exposure to the acidic environment, the control group exhibited the highest post-immersion surface roughness (0.1902 ± 0.0103 µm), which was significantly greater than those of all GO-containing specimens (*p* < 0.05). Among the low GO concentration groups, specimens containing 0.025 wt %, 0.05 wt %, and 0.1 wt % GO demonstrated moderate increases in surface roughness (0.1261 ± 0.0076 µm, 0.1023 ± 0.0006 µm, and 0.0877 ± 0.0042 µm, respectively) with no statistically significant difference between these groups (*p* > 0.05). In contrast, specimens incorporating higher GO concentrations exhibited substantially lower surface roughness values after acid immersion. The 0.5 wt % and 1.0 wt % GO groups showed surface roughness values of 0.0263 ± 0.0012 µm and 0.0429 ± 0.0040 µm, respectively, with no statistically significant difference between these concentrations (*p* > 0.05). Within each group, the control group (0 wt % GO) and specimens containing 0.025, 0.05, and 0.1 wt % GO showed a statistically significant increase in surface roughness after immersion compared to their baseline values (*p* < 0.05). In contrast, no statistically significant differences were observed between pre- and post-immersion surface roughness values in the 0.5 and 1.0 wt % GO groups (*p* > 0.05). These findings indicate that higher GO loadings, particularly at concentrations of 0.5 wt % and above, effectively suppressed acid-induced surface degradation of the TC100 resin.

**Figure 4:**
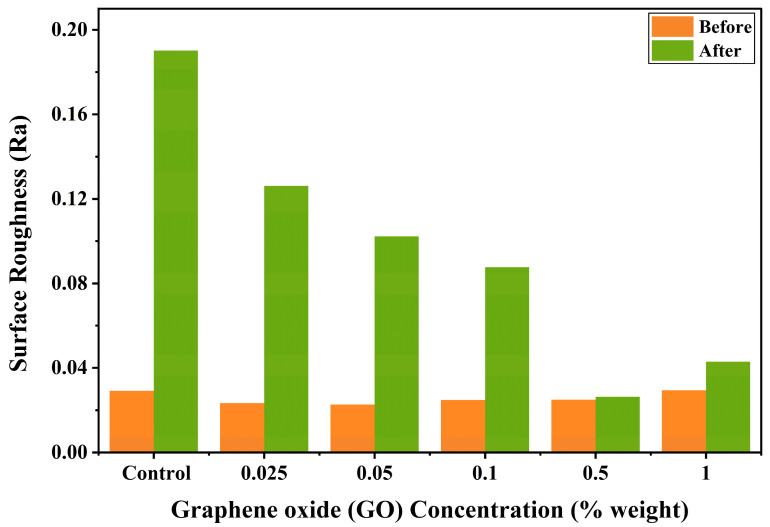
Comparative surface roughness of TC100 resin specimens containing varying concentrations of GO, measured before and after immersion in a simulated gastric acid solution (pH 2).

Figure 4 depicts the surface roughness (Ra) of TC100 resin specimens containing varying concentrations of GO (0, 0.025, 0.05, 0.1, 0.5 and 1.0 wt %) before and after immersion in a simulated gastric acid solution (pH 2). Prior to acidic exposure, all experimental groups exhibited low and relatively comparable surface roughness values, indicating minimal influence of GO incorporation on the initial surface topography. Following immersion, a marked increase in surface roughness was observed in all groups. The surface roughness of TC100 resin specimens demonstrated that the control group (0 wt % GO) showed statistically significant differences compared with all GO incorporated groups (*p* < 0.05). No statistically significant differences were observed among the 0.025, 0.05, and 0.1 wt % GO groups (*p* > 0.05). Similarly, no significant differences were found between the 0.5 and 1.0 wt % GO groups (*p* > 0.05). The magnitude of this increase was strongly dependent on GO concentration. The unmodified control group (0 wt % GO) demonstrated the highest post-immersion roughness, indicating extensive surface degradation. In contrast, specimens containing GO exhibited a concentration-dependent reduction in acid-induced surface roughening. The 0.025, 0.05, and 0.1 wt % GO groups showed progressively lower post-immersion roughness values compared with the control. The lowest surface roughness after acid exposure was recorded in the 0.5 wt % GO group, reflecting superior resistance to acidic erosion. Although a slight increase in surface roughness was observed at 1.0 wt % GO, this value remained substantially lower than that of the control group. Overall, these findings demonstrate that GO incorporation significantly enhances the resistance of TC100 resin to acid-induced surface degradation, with an optimal protective effect observed at a concentration of 0.5 wt % GO.

#### 3.2.2. Surface Hardness

**Figure 5:**
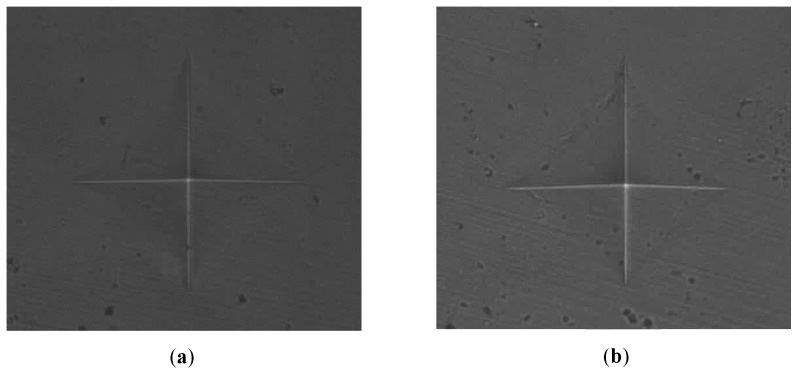
Representative optical micrographs of Vickers indentations on 0.5 wt % GO specimen (**a**) before and (**b**) after acid immersion at 45 h.

Figure 5 illustrates the comparative surface hardness of TC100 resin modified with 0.5 wt % GO before (a) and after (b) 45 h of acid immersion. Prior to exposure, the specimen exhibited a relatively smooth and uniform surface, indicative of higher surface hardness. Following acid immersion, a slight reduction in surface hardness was observed, as evidenced by subtle surface irregularities and microstructural changes. Nevertheless, compared with lower GO concentrations and the control group, the 0.5 wt % GO-modified resin demonstrated minimal degradation, confirming its superior resistance to acid-induced surface softening.

**Figure 6:**
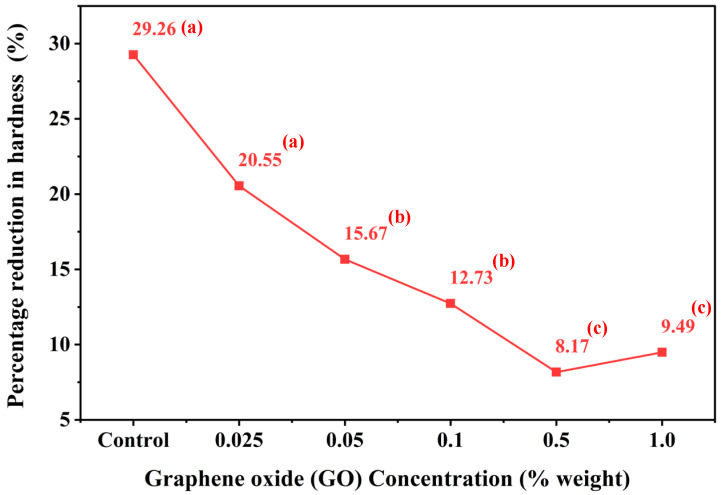
The graph illustrates the quantitative relationship between the percentage reduction in hardness and concentrations relative to the control. Different lowercase superscript letters indicate significant differences among groups (*p* < 0.05).

Figure 6 illustrates the quantitative relationship between the percentage reduction in surface hardness (%) and GO concentrations relative to the control group, both before and after immersion in a simulated gastric acid solution (pH 2). Inferential statistical analysis revealed no statistically significant difference in surface hardness reduction between the 0 wt % and 0.025 wt % GO groups (*p* > 0.05). Similarly, no significant differences were observed between the 0.05 wt % and 0.1 wt % GO groups (*p* > 0.05). In addition, the comparison between the 0.5 wt % and 1.0 wt % GO groups showed no statistically significant difference (*p* > 0.05). Collectively, these findings indicate that surface hardness responses remained statistically comparable within each respective GO concentration range (Figure 6).

Figure 6 presents the percentage reduction in surface hardness of TC100 resin as a function of GO concentration following acidic immersion. The unmodified control group exhibited the greatest decrease in surface hardness (29.26%). Progressive incorporation of GO resulted in a concentration-dependent reduction in hardness loss, with decreases of 20.55%, 15.67%, and 12.73% observed in the 0.025, 0.05, and 0.1 wt % GO groups, respectively. The minimum reduction in surface hardness was recorded at 0.5 wt % GO (8.17%), indicating optimal resistance to acid-induced degradation. A slight increase in hardness reduction was observed at 1.0 wt % GO (9.49%); however, this value remained substantially lower than that of the control group. These results confirm that GO incorporation significantly enhances the resistance of TC100 resin to acidic degradation, with the most pronounced protective effect observed at 0.5 wt % GO.

#### 3.2.3. Scanning Electron Microscopy (SEM) and Energy-Dispersive X-Ray Spectroscopy (EDS) Analysis

**Figure 7:**
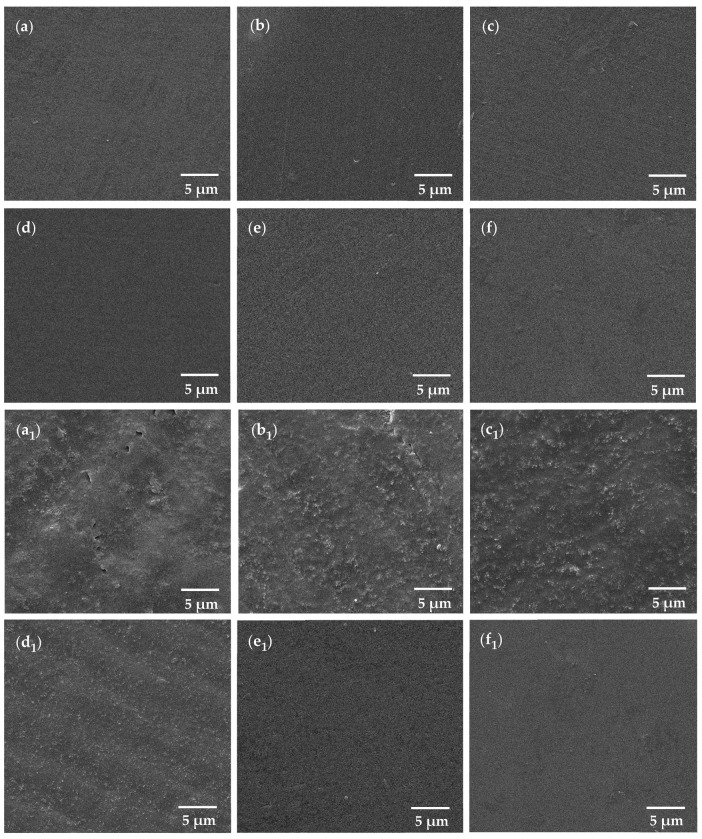
SEM image at 10,000× magnification of control group and GO incorporated in experimental resin: TC100 (control) (**a**,**a_1_**), 0.025 wt % GO incorporated in TC100 (**b**,**b_1_**), 0.05 wt % GO incorporated in TC100 (**c**,**c_1_**), 0.1 wt % GO incorporated in TC100 (**d**,**d_1_**), 0.5 wt % GO incorporated in TC100 (**e**,**e_1_**), 1.0 wt % GO incorporated in TC100 (**f**,**f_1_**). (**a_1_**–**f_1_**) were specimens subsequent to immersion in a simulated gastric acid solution (pH 2).

Figure 7 presents SEM micrographs obtained at 10,000× magnification, illustrating the surface morphology of TC100 resin specimens before and after immersion in a simulated gastroesophageal reflux environment for the control group and GO-modified experimental groups.

SEM analysis conducted prior to acid immersion (Figure 7a–f) demonstrated that the GO-modified specimens exhibited smooth and uniform surface morphologies, without evident agglomeration or irregular clustering of GO, indicating effective and homogeneous dispersion of GO nanosheets within the polymer matrix via ball milling. The control groups (0 wt % GO; a, a_1_) exhibited the most severe surface deterioration following acid immersion. The post-immersion surfaces showed pronounced roughness, with clear evidence of acid penetration into the subsurface regions and the presence of distinct surface crack lines, indicating substantial acid-induced degradation. Specimens containing 0.025 wt % GO (b, b_1_) displayed noticeable surface alterations after immersion, characterized by multiple large etched regions distributed across the surface. Surface crack lines remained evident, suggesting that this GO concentration provided limited protection against acid-induced surface damage. Similarly, the 0.05 wt % GO group (c, c_1_) exhibited extensive etched areas with a more pronounced and patterned etching morphology, reflecting continued susceptibility to acidic attack. In contrast, specimens incorporating 0.1 wt % GO (d, d_1_) demonstrated comparatively reduced surface degradation after immersion. The surface morphology was characterized by numerous small and finely distributed etched features, and no surface crack lines were observed, indicating enhanced resistance to acid-induced surface damage at this concentration. Specimens modified with higher GO concentrations, specifically 0.5 wt % and 1.0 wt % GO (e, e_1_, f, and f_1_), exhibited predominantly smooth surface morphologies following acid immersion. The post-immersion surfaces of these groups closely resembled those observed prior to immersion and were comparable to the untreated control surface before acid exposure, suggesting effective suppression of acid-induced surface degradation at higher GO loadings.

**Figure 8:**
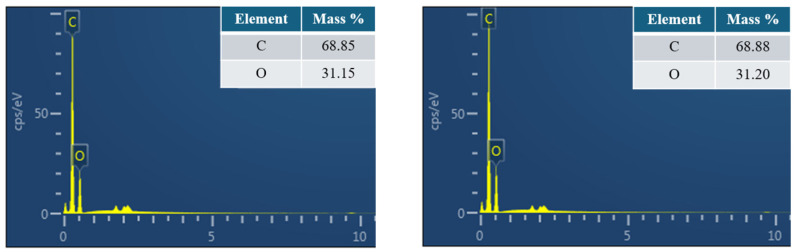
Energy-dispersive X-ray spectroscopy (EDS) spectra illustrating the elemental composition of TC100 control and TC100 modified with 1.0 wt % GO.

EDS analysis revealed that the TC100 control group exhibited a carbon-to-oxygen (C:O) atomic ratio of 68.85:31.15, while the TC100 specimen incorporating 1.0 wt % GO showed a comparable C:O ratio of 68.88:31.20 (Figure 8). No discernible difference in elemental composition was observed between the control and GO-modified groups. This similarity is attributed to the relatively low GO loading within the resin matrix. Given that the provisional resin matrix is predominantly composed of carbon-based polymer chains and that GO consists of single-layer carbon atoms decorated with oxygen-containing functional groups, the incorporation of GO at this concentration did not produce a measurable change in the overall elemental profile. Consequently, carbon and oxygen were the only elements detected in both groups. Although the EDS elemental composition remains relatively unchanged, SEM reveals notable differences. GO acts as an effective physical barrier, likely due to its influence on microstructural morphology.

## 4. Discussion

The present study aimed to evaluate the effect of GO incorporation on the surface properties of 3D-printed provisional restorations under simulated gastric acid conditions. The most significant finding was that the addition of GO effectively enhanced the acid resistance of the resin matrix, with the 0.5 wt % concentration demonstrating the optimal balance of surface roughness and hardness stability. Based on these results, the first null hypothesis, which stated that varying GO concentrations would not affect surface properties, was rejected.

The superior acid resistance of the modified specimens can be attributed to the multifaceted protective properties of GO. Its sp^2^-hybridized carbon backbone possesses high bond dissociation energies, making it chemically inert to protonic attack (H^+^) typical of gastric fluid [59]. Beyond this intrinsic stability, oxygenated surface groups create a hydration shell that serves as a physical barrier, hindering acid penetration [59,60]. This barrier is strengthened by electrostatic repulsion against hydronium ions (H_3_O^+^) and the acid-triggered aggregation of GO sheets, which reduces the surface area available for degradation [61]. Collectively, these factors act synergistically to block acid infiltration into the resin matrix, ensuring the durability of the restoration. Interestingly, a dose-dependent improvement was observed up to 0.5 wt %, beyond which no further significant enhancement was noted. At the higher concentration of 1.0 wt %, the slight decline in surface properties may be attributed to the agglomeration of GO nanosheets within the resin matrix, which can act as stress concentration points and disrupt the polymer network efficiency [62].

Previous studies have reported that homogeneous dispersion of GO within polymer matrices enhances resistance to chemical and corrosive degradation by inhibiting the diffusion of acidic solutions [32,33,34,35,36,39]. This study demonstrates that incorporation of GO significantly influences the acid resistance of the TC100 resin, consistent with previous reports that GO enhances the chemical stability of polymer matrices through its barrier effect and strong interfacial interactions with the polymer network [63]. The SEM showed severe surface roughening, crack formation, and subsurface penetration observed in the unmodified control indicate that the polymer matrix is highly susceptible to acidic degradation, which is typically associated with hydrolytic attack and polymer chain scission under acidic conditions [64]. Previous studies have consistently demonstrated the presence of an optimal GO loading, generally within low concentration ranges (0.05–0.1 wt %), at which homogeneous nanosheet dispersion maximizes barrier efficiency and corrosion resistance [65]. From this study, low GO loadings (0.025–0.05 wt %), only limited improvement was observed, suggesting that insufficient nanosheet content and possible incomplete dispersion may fail to effectively block acid diffusion pathways [66]. However, at 0.1 wt % GO, the absence of crack lines and the presence of finely distributed etched features indicate improved structural integrity, implying better dispersion and stronger interfacial bonding between GO and the resin matrix, as reported in GO/polymer nanocomposite systems [67]. At higher concentrations (0.5–1.0 wt %), the smooth post-immersion surfaces suggest that GO nanosheets formed a protective barrier that reduced acid permeation and inhibited polymer degradation, which can be explained by the high aspect ratio and layered structure of GO creating a tortuous diffusion pathway for corrosive media [68]. This study suggests that the incorporation of GO improves the acid resistance of the resin at all investigated concentrations; however, the performance is strongly dependent on dispersion within the matrix [67,69]. The incorporation of GO nanoparticles at 0.1% and 0.5% resulted in increased microhardness and reduced surface roughness of the resin-modified glass ionomer cement (RMGIC), without producing significant changes in flexural strength or elastic modulus [70]. An optimal GO loading of 0.7 wt % was identified, at which the epoxy resin coating exhibited the most significant improvements in mechanical performance and erosion resistance [71]. In this study, the 0.5 wt % GO specimen exhibited the most homogeneous morphology, indicating optimal nanosheet distribution and effective interfacial interaction with the polymer network. This uniform dispersion likely enabled the formation of a continuous barrier that restricted acid diffusion and reduced structural degradation. Although every GO-modified group demonstrated resistance to acidic exposure compared with the unmodified resin, the 0.5 wt % GO group showed the highest resistance, as evidenced by the smallest change in hardness after immersion. This minimal hardness variation indicates better preservation of the polymer structure and mechanical integrity, confirming that 0.5 wt % represents the most effective concentration for enhancing acid durability.

Although commercial GO is available, in-house synthesis via the Hummers’ method was employed in this study to ensure strict control over purity, oxidation degree, and particle size distribution. Commercial GO products often exhibit batch-to-batch variability, which can detrimentally affect the optical and mechanical consistency of dental resins. Furthermore, the in-house synthesis allowed for thorough purification to remove residual ions, ensuring the biocompatibility required for intraoral use, while also offering a cost-effective solution for potential future clinical upscaling. The successful synthesis and functionalization of GO, as comprehensively confirmed by our structural and chemical characterizations (XRD, FTIR, and Raman spectroscopy), provided the essential oxygen-containing groups. These functional groups not only expanded the interlayer spacing but also facilitated the uniform dispersion of GO nanosheets within the TC100 resin matrix, which is a critical prerequisite for establishing an effective acid-resistant barrier [46].

The surface roughness analysis further corroborated the barrier efficacy of GO. While lower concentrations offered marginal improvements, incorporating GO at 0.5 wt % and above established a protective threshold, effectively mitigating acid-induced surface deterioration to levels comparable to the pre-immersion state. This protective mechanism directly translated to the preservation of mechanical integrity. The progressive retention of surface hardness corresponding to increased GO loading underscores the material’s ability to withstand hydrolytic degradation, with the 0.5 wt % formulation demonstrating the optimal balance between matrix reinforcement and acid resistance. Consequently, the second null hypothesis was partially rejected; while the unmodified control and low-GO concentration groups exhibited substantial acid-induced alterations, the materials modified with 0.5 and 1.0 wt % GO effectively maintained their baseline surface properties following simulated GERD exposure. This outcome confirms that a threshold concentration of 0.5 wt % is required to achieve optimal acid durability and structural stability under these aggressive conditions. The improvement in microhardness observed at GO loadings of 0.3–0.7 wt % suggests that an optimal filler concentration plays a critical role in reinforcing the coating matrix of temporary materials. At this range, GO likely promotes effective stress transfer and restricts polymer chain mobility, thereby enhancing the overall mechanical performance [72]. GO exhibits exceptional impermeability due to its densely packed carbon lattice, high π-electron density that repels incoming molecules, remarkable mechanical strength, and atomically thin structure [73]. The observed behavior is closely related to the extraordinary mechanical integrity of graphene. Its high C–C bond energy (4.9 eV) together with an intrinsic strength of 43 N/m provides a densely packed atomic lattice that resists structural deformation and defect formation. As a consequence, graphene functions as an atomically thin yet effectively impermeable membrane, preventing molecular transport across the layer despite its minimal thickness [73].

GO stands out for its exceptional strength and unique properties, particularly its impressive resistance to acidic environments [32,33,34,35,36,39]. Despite these advantages, its application in dentistry has been largely underexplored. This study bridges that gap by successfully incorporating GO into 3D-printed provisional restorations. Our findings confirm that GO acts as an effective reinforcement agent, significantly enhancing both acid resistance and mechanical performance. These results highlight GO as a promising candidate for next-generation dental materials, paving the way for future advancements in material stability and clinical durability.

However, there are some limitations to this study that must be acknowledged. First, the experiment was conducted using a static acid immersion model, which does not fully replicate the complex, dynamic oral environment involving chewing forces, saliva flow, and temperature changes. Second, the potential impact of GO’s naturally dark color on the esthetics of the restoration was not evaluated. Future research should therefore investigate the color stability and translucency to ensure acceptable clinical appearance. Finally, biocompatibility testing was not included in this phase; this is an essential next step to confirm the biological safety of the material before clinical application.

## 5. Conclusions

The incorporation of GO significantly improved the surface integrity and acid resistance of 3D-printed provisional resin under acidic conditions. Among the concentrations evaluated, 0.5 wt % GO was identified as the optimal loading, effectively preserving surface roughness and hardness near baseline values while outperforming both the control and other experimental groups. SEM analysis revealed that specimens containing 0.5 wt % GO maintained predominantly smooth surface morphologies after acid immersion, closely resembling the untreated control prior to exposure. This observation suggests that GO functions as an effective physical barrier, thereby limiting acid penetration and surface degradation. Consequently, the 0.5 wt % GO loading mitigates acid-induced deterioration and results in a comparatively lower reduction in surface hardness (8.17%). Collectively, these findings underscore the potential of GO-modified 3D-printed resin as a promising material for fabricating durable provisional restorations, particularly for patients exposed to highly acidic oral environments, including those with GERD.

## Figures and Tables

**Table 1:** Materials and their compositions used in the study.

Materials	Chemical Compositions	Manufacturer
**Graphene Oxide Synthesis**		
Graphite (282863)	Carbon	Sigma-Aldrich company (Missouri, St. Louis, USA)
Sodium Nitrate (NaNO_3_) (grade AR, QS5248-1)	-	QReC™ (New Zealand)
Sulfuric Acid (H_2_SO_4_) (grade AR, QS7064-1)	-	QReC™ (New Zealand)
Potassium Permanganate (KMnO_4_) (grade AR, QP5219-1-0500)	-	QReC™ (New Zealand)
Hydrochloric acid (HCl) (grade AR, QH8040-1-2501)	-	QReC™ (New Zealand)
Hydrogen peroxide (H_2_O_2_)	-	Samchun Chemical (Gangnam-gu, Seoul, Republic of Korea)
**Gastric acid preparation**		
Sodium Chloride (NaCl) (grade GR, CAS number 7647-14-5)	-	Merck KGaA (Darmstadt, Germany)
Pepsin (CAS: 9001-75-6)	-	Bendosen (Seremban, Negeri Sembilan, Peninsular Malaysia)
Hydrochloric acid (HCl) (grade AR, QH8040-1-2501)	-	QReC™ (New Zealand),
**Resin Specimen**		
TC100 Temporary Crown & Bridge Resin (TC100) A3—0.5 KG Lot number: N/A (Product code: EAN 6922572203457)	Urethane acrylate resin 40–50 wt % Monomer 20–40 wt % Photo initiators 3–5 wt % Color pigment 2–5 wt %	Shenzhen Esun Industrial Company (Shenzhen, China)

**Table 2:** Mean ± standard deviation of surface roughness values (Ra, µm) in the experimental group before and after acid immersion (n = 10).

TC 100 Temporary Crown & Bridge Resin (TC100)	Concentration of GO (wt %)					
	Control	0.025	0.05	0.1	0.5	1.0
Before	0.0291 ± 0.0057 ^(a,1)^	0.0232 ± 0.0005 ^(a,1)^	0.0226 ± 0.0003 ^(a,1)^	0.0247 ± 0.0043 ^(a,1)^	0.0249 ± 0.0025 ^(a,1)^	0.0294 ± 0.0064 ^(a,1)^
After	0.1902 ± 0.0103 ^(a,2)^	0.1261 ± 0.0076 ^(b,2)^	0.1023 ± 0.0006 ^(b,2)^	0.0877 ± 0.0042 ^(b,2)^	0.0263 ± 0.0012 ^(c,1)^	0.0429 ± 0.0040 ^(c,1)^

The same lowercase superscript letter (in rows) indicates mean values without statistically significant differences (*p* > 0.05). The same number (in columns) indicates mean values without statistically significant differences (*p* > 0.05).

## Data Availability

The original contributions presented in the study are included in the article; further inquiries can be directed to the corresponding authors.
